# *Ex situ* Normothermic Split Liver Machine Perfusion: Protocol for Robust Comparative Controls in Liver Function Assessment Suitable for Evaluation of Novel Therapeutic Interventions in the Pre-clinical Setting

**DOI:** 10.3389/fsurg.2021.627332

**Published:** 2021-02-17

**Authors:** Joseph A. Attard, Daniel-Clement Osei-Bordom, Yuri Boteon, Lorraine Wallace, Vincenzo Ronca, Gary Reynolds, M. T. P. R. Perera, Ye Htun Oo, Hynek Mergental, Darius F. Mirza, Simon C. Afford

**Affiliations:** ^1^National Institute for Health Research (NIHR) Birmingham Biomedical Research Centre, University Hospitals Birmingham National Health Service (NHS) Foundation Trust, University of Birmingham, Birmingham, United Kingdom; ^2^Centre for Liver and Gastrointestinal Research, Institute of Immunology and Immunotherapy, University of Birmingham, Birmingham, United Kingdom; ^3^Liver Unit, Queen Elizabeth Hospital, University Hospitals Birmingham National Health Service (NHS) Foundation Trust, Birmingham, United Kingdom; ^4^Centre for Rare Disease, European Reference Network Centre (ERN RARE-LIVER), Hamburg, Germany

**Keywords:** normothermic, liver function, organ preservation, split liver technique, machine perfusion

## Abstract

**Background:** *Ex situ* donor liver machine perfusion is a promising tool to assess organ viability prior to transplantation and platform to investigate novel therapeutic interventions. However, the wide variability in donor and graft characteristics between individual donor livers limits the comparability of results. We investigated the hypothesis that the development of a split liver *ex situ* machine perfusion protocol provides the ideal comparative controls in the investigation of machine perfusion techniques and therapeutic interventions, thus leading to more comparable results.

**Methods:** Four discarded human donor livers were surgically split following identification and separation of right and left inflow and outflow vessels. Each lobe, on separate perfusion machines, was subjected to normothermic perfusion using an artificial hemoglobin-based oxygen carrier solution for 6 h. Metabolic parameters as well as hepatic artery and portal vein perfusion parameters monitored.

**Results:** Trends in hepatic artery and portal vein flows showed a general increase in both lobes throughout each perfusion experiment, even when normalized for tissue weight. Progressive decreases in perfusate lactate and glucose levels exhibited comparable trends in between lobes.

**Conclusion:** Our results demonstrate comparability between right and left lobes when simultaneously subjected to normothermic machine perfusion. In the pre-clinical setting, this model provides the ideal comparative controls in the investigation of therapeutic interventions.

## Introduction

The main purpose of *ex situ* donor liver machine perfusion has been the development of superior modality of organ preservation to conventional static cold storage as well as a method to assess organ viability prior to transplantation (1). In the United Kingdom, between April 2018 and March 2019, 15% of donor livers retrieved were not transplanted, representing a significant pool of potentially viable grafts (2) A recent randomized controlled clinical trial demonstrated that normothermic machine liver perfusion (NMLP) reduced discard rates of donor organs when compared to static cold storage, without jeopardizing transplant outcomes (3, 4). A number of perfusion devices are currently available. The principal components include a blood reservoir, centrifugal pump, oxygen concentrator, heat exchanger and a circuit which continuously pumps perfusate through the liver via the organ's inflow vessels (hepatic artery and/or portal vein) and recirculates this following drainage from the inferior vena cava (1). These systems allow for extraction of perfusate for blood gas analysis, thus enabling real-time monitoring of oxygen and carbon dioxide levels, acid-base homeostasis as well as glucose levels (1) All these parameters have been described as markers for organ viability during machine perfusion (5). Several pre-clinical and clinical non-randomized studies have investigated different perfusion modalities with variations in perfusion temperature, perfusate composition, perfusion duration, vessel cannulation as well as other technical considerations (1).

The technology provides a unique opportunity for assessing liver metabolic and synthetic function. This has been recognized by pre-clinical studies investigating the effect of novel therapeutic interventions on machine perfused donor livers (6, 7). However, donor livers available for research in machine perfusion have been, so far, in short supply. Furthermore, the inherent variation in donor characteristics and liver quality that exist between different livers have limited their capacity as suitable comparative controls and, therefore, the interpretability of data from small series of whole organ perfusions (8).

Splitting of the donor liver is a well-established strategy used in transplantation to increase organ availability by allowing the same organ to be “shared” between two recipients (9). The surgical technique splits the donor organ into two separate independently-functioning units (9). A pre-clinical study by Huang et al. reported that, during *ex situ* subnormothermic machine perfusion, split livers demonstrated comparable perfusion and functional characteristics, providing a controlled comparison between split lobes, thus allowing each liver to act as its own internal control (10). However, this has yet to be demonstrated in a normothermic machine perfusion model. NMLP has been shown to enable the functional assessment and viability testing of donor livers prior to transplantation in a near-physiological environment (5, 11). In the context of scientific research, this makes NMLP a promising pre-clinical platform for the investigation of therapeutics and mechanistic studies. We therefore sought to develop a normothermic machine perfusion protocol for the application of the split liver model to pre-clinical research. Our hypothesis was that individual lobes from the same liver would recover and function similarly to one another when subjected to *ex situ* end-ischaemic NMLP. In addition to providing more liver units for perfusion experiments, the protocol enables each liver to act as its own internal control, thus eliminating the inherent heterogeneity of whole organ perfusions.

## Materials and Methods

### Study Design

This study was designed to investigate and compare the performance of right and left lobes from the same human liver. Each lobe has their own inflow and outflow vessels as well as bile drainage, during *ex situ* end-ischaemic NMLP on separate perfusion devices, thereby demonstrating their suitable utilization as comparative controls in the pre-clinical setting. The primary endpoints were assessment of liver function and evaluation of perfusion parameters. The perfusion machines used for this study both were Liver Assist devices (Organ Assist, Groningen, The Netherlands).

### Donor Liver Source and Selection

Four donor livers were included in this study, resulting in a total of eight perfusions. All donor livers included in this study were originally retrieved with the primary intention for transplantation as per the policy of the National Health Service Blood and Transplant (NHSBT). The organs were subsequently rejected for transplantation by all UK liver transplant centers and, following that, were offered nationally for research by NHSBT. The authors had no influence in the process of declining donor livers. This was done by the transplant surgeons at each center. Informed consent for research use of donor organs was obtained by specialist nurses in organ donation from the donor's next of kin during informed consent for organ donation. Authorisation for research was mediated by each centre's specialist nurse in organ donation. All methods described were performed in accordance with NHSBT guidelines and regulations. Study ethical approval was obtained from the London-Surrey Borders National Research Ethics Service (Reference Number 13/LO/1926) and the NHSBT ethics committee (Reference Number 06/Q702/61). No organs used in this study originated from executed prisoners.

Donor liver exclusion criteria for this study were: gross macroscopic appearance indicative of moderate or severe steatosis; asymmetric or poor perfusion demonstrated during organ retrieval; presence of hepatic malignancy; organ subjected to machine perfusion prior to being discarded.

### Liver Splitting Protocol

**Figure 1:**
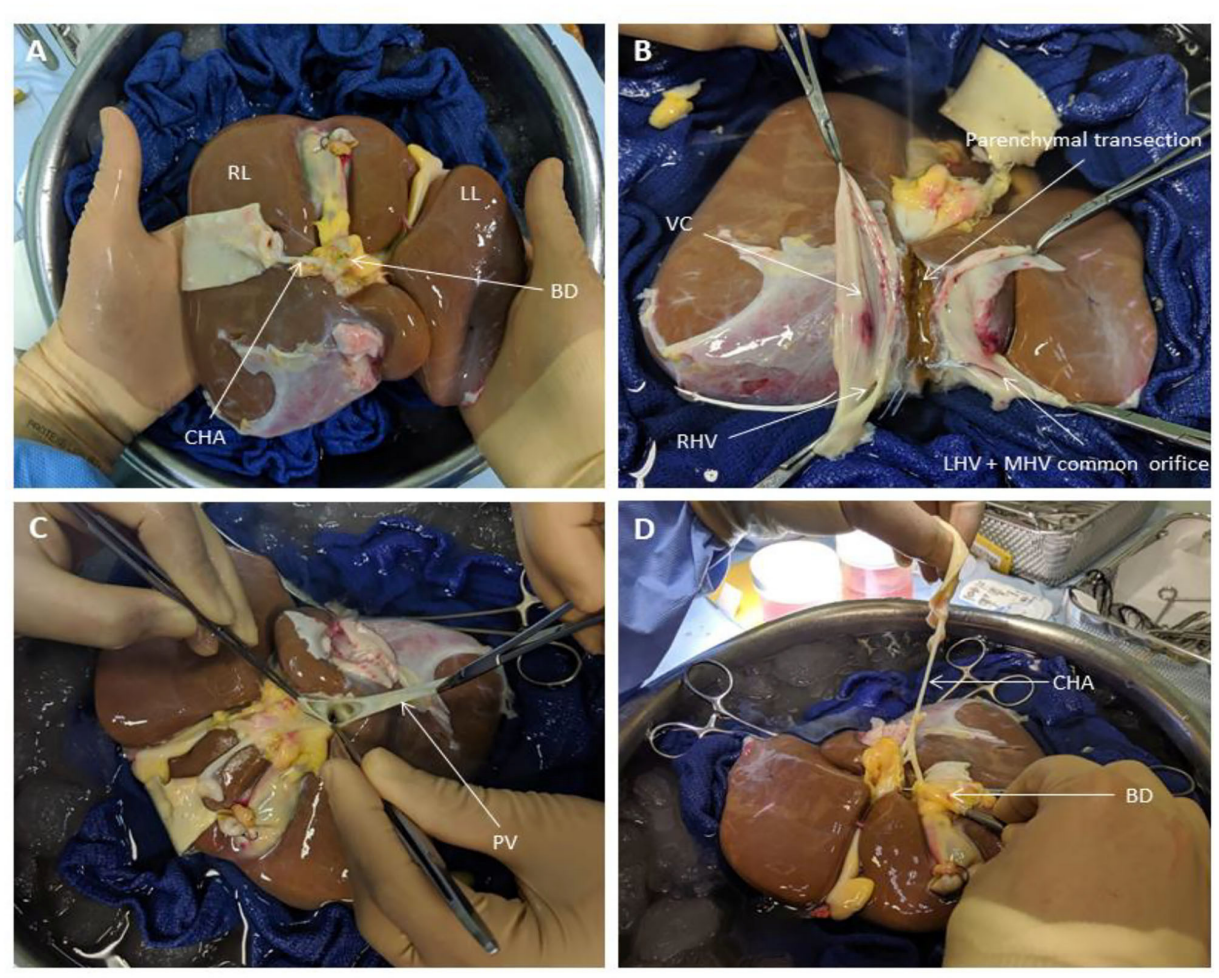
Liver Splitting Procedure: **(A)** Inspection of liver hilum **(B)** Opening inferior vena cava to identify hepatic venous drainage **(C)** Identification of portal vein branches **(D)** Identification of hepatic arterial branches and hepatic duct transection. BD, bile duct; CHA, common hepatic artery; LHV, left hepatic vein; LL, left lobe; MHV, middle hepatic vein; PV, portal vein; RL, right lobe; RHV, right hepatic vein; VC, vena cava.

**Figure 2:**
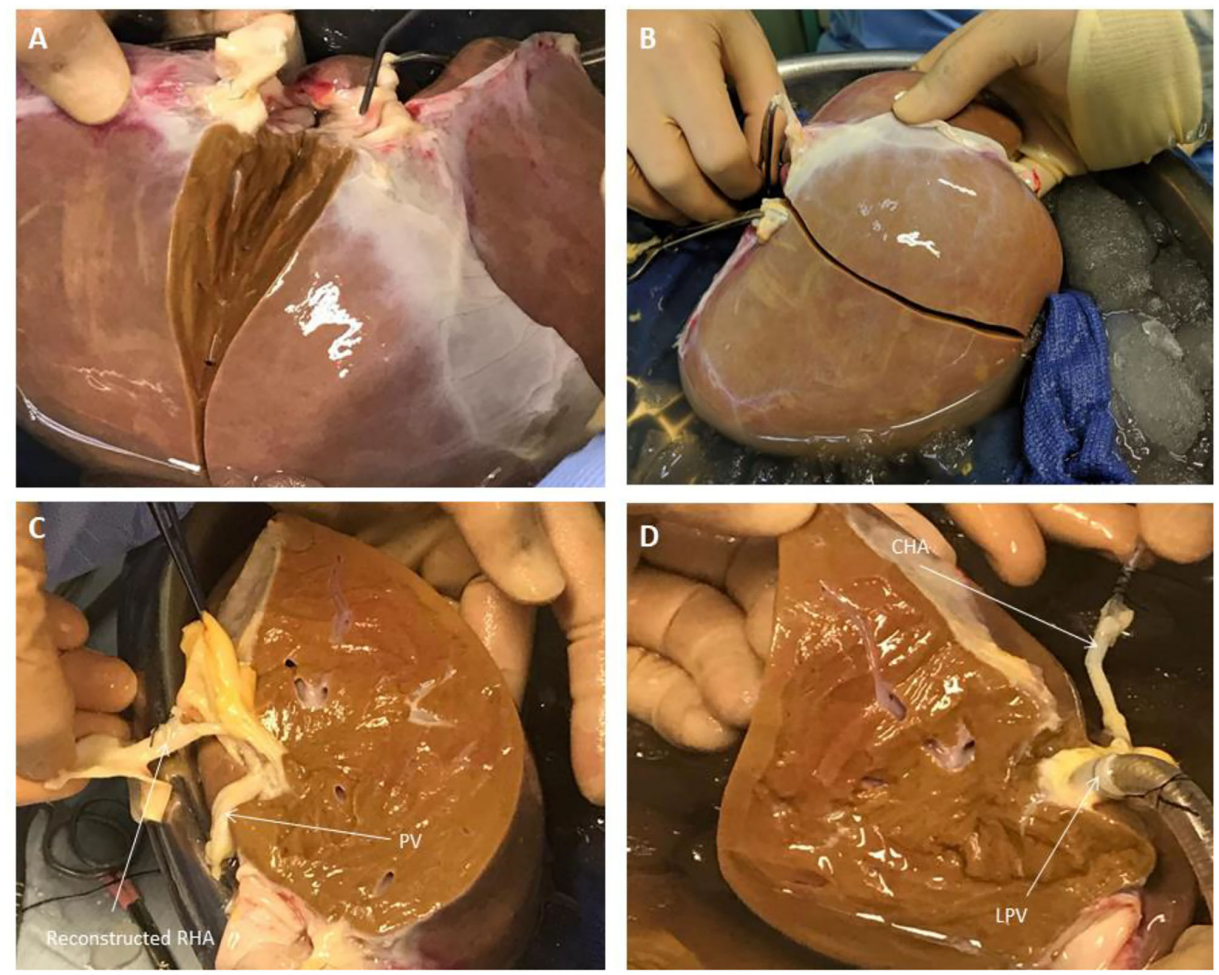
Liver Splitting Procedure: **(A)** Parenchymal transection across gallbladder bed **(B)** Parenchymal transection complete **(C)** Reconstruction of hepatic artery using donor celiac artery trunk **(D)** Cannulation of hepatic artery and portal vein branches. CHA, common hepatic artery; LPV, left portal vein; PV, portal vein; RHA, right hepatic artery.

During transportation to our center, the organs were preserved in University of Wisconsin fluid at hypothermic temperatures in ice as per current standard clinical practice for static cold storage in the United Kingdom. Donor livers were split while in cold storage prior to commencement of perfusion in order to ensure that both lobes were subjected to similar ischaemic times and assessed simultaneously when placed in the perfusion devices. Upon receipt by our center, the liver was initially cleared of excess tissue in order to assess the quality of the organ. This also allowed for assessment of the organ's outflow, with respect to the hepatic veins and post-hepatic inferior vena cava, as well as the inflow, in terms of the hepatic arterial tree and portal vein (Figure 1). Since the perfusion device allows for open drainage of the perfusate from the organ's outflow via the post-hepatic inferior vena cava, the latter was incised in order to create two separate patches with direct visualization of left, middle and right hepatic veins (Figure 1). This was the first step in determining the line of demarcation for parenchymal division. For the purposes of this study, we sought to divide right and left halves of the liver along Cantlie's line—an extrapolated line, used when planning right or left hepatectomies (liver resections), extending from the middle of the post-hepatic inferior vena cava across the diaphragmatic (superior) surface of the liver to the point where the fundus of the gallbladder on the inferior surface typically contacts the antero-inferior margin of the liver (Figure 2). This approximates the plane of the middle hepatic vein and demarcates the right and left lobes of the liver.

Once the vena cava was opened, the liver hilum was cleared further of excess tissue in order to identify the left and right branches of the portal vein and hepatic artery, respectively (Figure 1). Particular attention was paid to identification of the arterial branch supplying segment 4 of the liver as this would influence the level of division of the branches during splitting. The left and right hepatic ducts were also identified to ensure that each half would have adequate biliary drainage (Figure 1). A cholecystectomy was performed, and the cystic duct identified and ligated. Following satisfactory identification of all relevant anatomy, the vessels and bile duct were divided as follows. The hepatic arterial tree was divided such that the main trunk was retained by the left lobe of the liver with the right lobe keeping the right hepatic branch, anatomy-permitting. The right lobe retained the main trunks of the portal vein and bile duct with the left halve keeping its respective branches. At this point, parenchymal division was performed and completed along Cantlie's line (Figure 2).

If vessel length and diameter was deemed to be inadequate for safe cannulation of either lobe, particularly with respect to the isolated branches, then a decision was made to reconstruct the vessels to enable proper positioning of the cannulas for perfusion. This was done by fashioning a conduit using native vessels for extra length in one of two ways: (1) resecting the distal portion of the main arterial or venous trunk retained by either lobe, which was then anastomosed onto the branch of the other; (2) utilizing the length of donor iliac vessels for the reconstruction (received with the donor liver for the purposes of arterial or venous reconstruction if required in the setting of transplantation) (Figure 2). Which technique was adopted was dependent on the conduit length required as well as the caliber of the vessels to be anastomosed together. The hepatic arteries were cannulated with 10−14 F cannulas. Each portal vein was cannulated with a 24 F cannula.

### *Ex-situ* Perfusion Protocol

Both perfusion devices were primed with our previously described (12) perfusion protocol using Hemopure [HBOC-201, hemoglobin glutamer-250 (bovine); HBOC-201, Hemoglobin Oxygen Therapeutics LLC, Cambridge, MA] instead of packed red blood cells as the oxygen carrier (Supplementary Material). The former is a polymerised bovine hemoglobin-based acellular oxygen carrier of low immunogenicity and an oxygen-carrying capacity similar to that of human hemoglobin at normothermic temperatures. Its efficacy as an alternative to blood-based machine perfusion fluid has been demonstrated in pre-clinical and clinical studies (12, 13).

**Figure 3:**
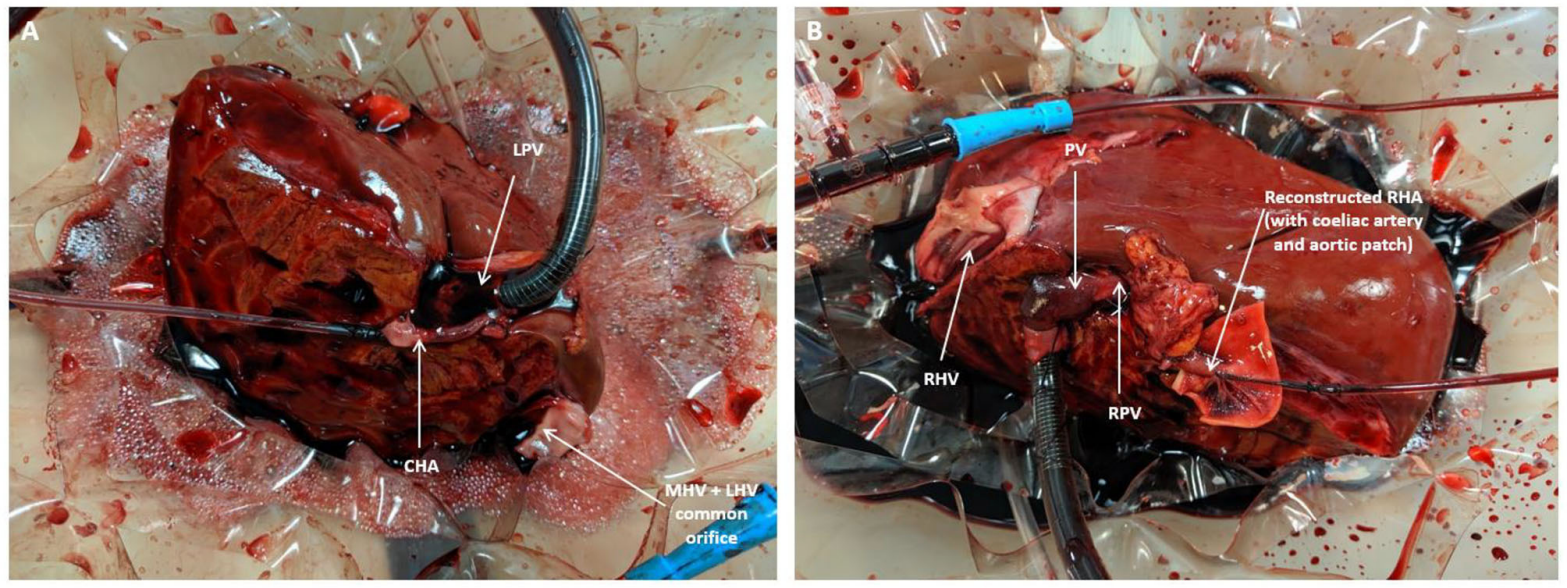
Split lobe perfusion: **(A)** Left lobe **(B)** Right lobe. CHA, common hepatic artery; LPV, left portal vein; MHV, middle hepatic vein; PV, portal vein; RHA, right hepatic artery; RPV, right portal vein.

The pre-perfusion weight of each lobe was recorded post-splitting. The arterial and portal venous supplies were cannulated with each lobe positioned inside the organ reservoir such that the open drainage from the hepatic veins could be visualized directly. Perfusion was commenced at 36–37°C with oxygenated pulsatile flow and non-pulsatile flow in the hepatic artery and PV, respectively. Perfusion of each lobe was commenced within five to 15 min of each other (Figure 3). Perfusion pressures and flow parameters were monitored continuously. An epoprostenol infusion pump was connected to each perfusion circuit and commenced at an initial rate of 4 ml/h. The rate of prostaglandin infusion was adjusted according to the flow readings to maintain physiological parameters. Hepatic artery pressures were maintained at 60–70 mm Hg while portal vein pressures were maintained at 10 mmHg. Oxygen supply was adjusted in order to maintain a perfusate oxygen partial pressure >10 kPa in the arterial circuit. Serial perfusate samples were analyzed in real-time using the Cobas b 221 point of care system (Roche Diagnostics, USA) Blood Gas Analyser in order to monitor metabolic parameters including oxygen partial pressures, lactate and glucose levels. These parameters have been previously described as appropriate methods of monitoring liver function and viability during the perfusion process (5). Each lobe underwent NMLP for a total of 6 h. For histological analysis, core needle biopsies from each lobe were obtained at the beginning and end of perfusion, fixed in formalin and embedded in paraffin. These biopsies were subsequently stained with haematoxylin and eosin for conventional examination and periodic acid schiff (PAS) for glycogen content and distribution.

### Statistical Analysis

Data analysis was carried out using Prism 7 (GraphPad Inc., CA). Continuous data at each timepoint was compared using Wilcoxon signed-rank test. Statistical significance was set at p <0.05. Data is presented as per lobar mass as well as following normalization of pre-perfusion lobar weight per gram of tissue.

## Results

### Donor Liver Characteristics

Four livers were included in the study. Information relating to donor characteristics and liver characteristics can be found in (Table 1). Right lobe mass was consistently significantly higher than their left lobe counterparts.

**Table 1:** Donor liver demographics and characteristics.

	**Liver 1**	**Liver 2**	**Liver 3**	**Liver 4**
Donor age (years)	31	75	57	73
Gender	Male	Male	Female	Female
DBD/DCD	DCD	DBD	DCD	DBD
Cold ischaemia time (min)	902	799	1,108	1,026
**Weight (g)**				
Right lobe	715	1,120	957	739
Left lobe	488	851	851	363

*DBD, donor after brainstem death; DCD, donor after cardiac death*.

### Assessment of Liver Function

**Figure 4:**
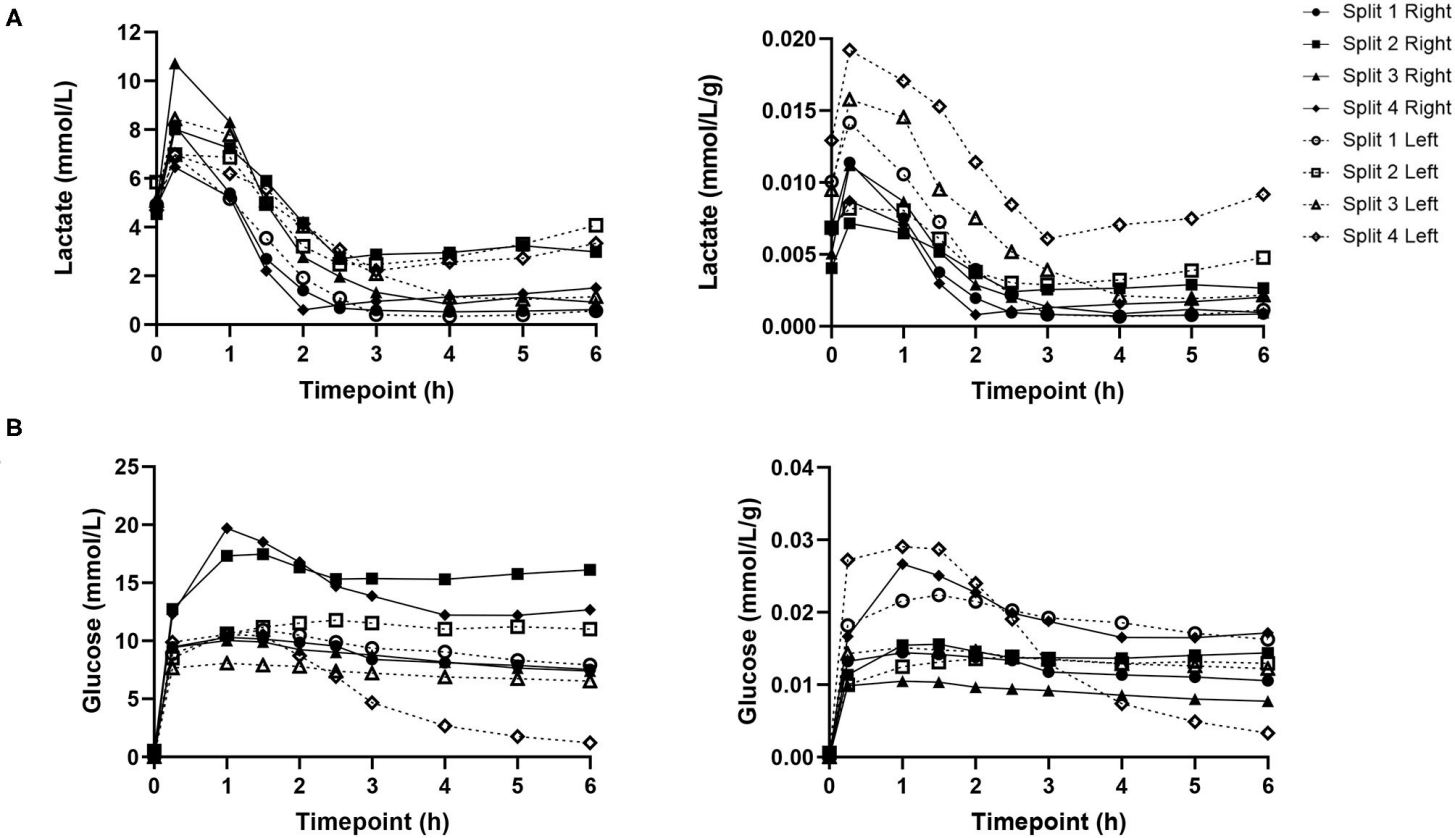
Perfusate lactate **(A)** and glucose **(B)** levels for each individual split lobe over 6 h of end-ischaemic normothermic machine perfusion.

Lactate levels were comparable at the start of perfusion, T0, and decreased significantly in all lobes up until the end of the perfusion experiment, T6 (Figure 4). Trends in perfusate lactate clearance were similar in both lobes. When normalized for pre-perfusion tissue weight, perfusate lactate levels tended to be higher in the left lobe. However, the rate of reduction in lactate levels was observed to be similar in both lobes across all perfusion experiments. Interestingly, in two of the four livers, an increase in perfusate lactate was observed in both lobes at the same timepoint during the perfusion experiment. Perfusate glucose levels were initially high and trended downwards during the course of the experiment. Lactate and glucose levels were comparable at all timepoints (Figure 4).

### Perfusion Hemodynamics

**Figure 5:**
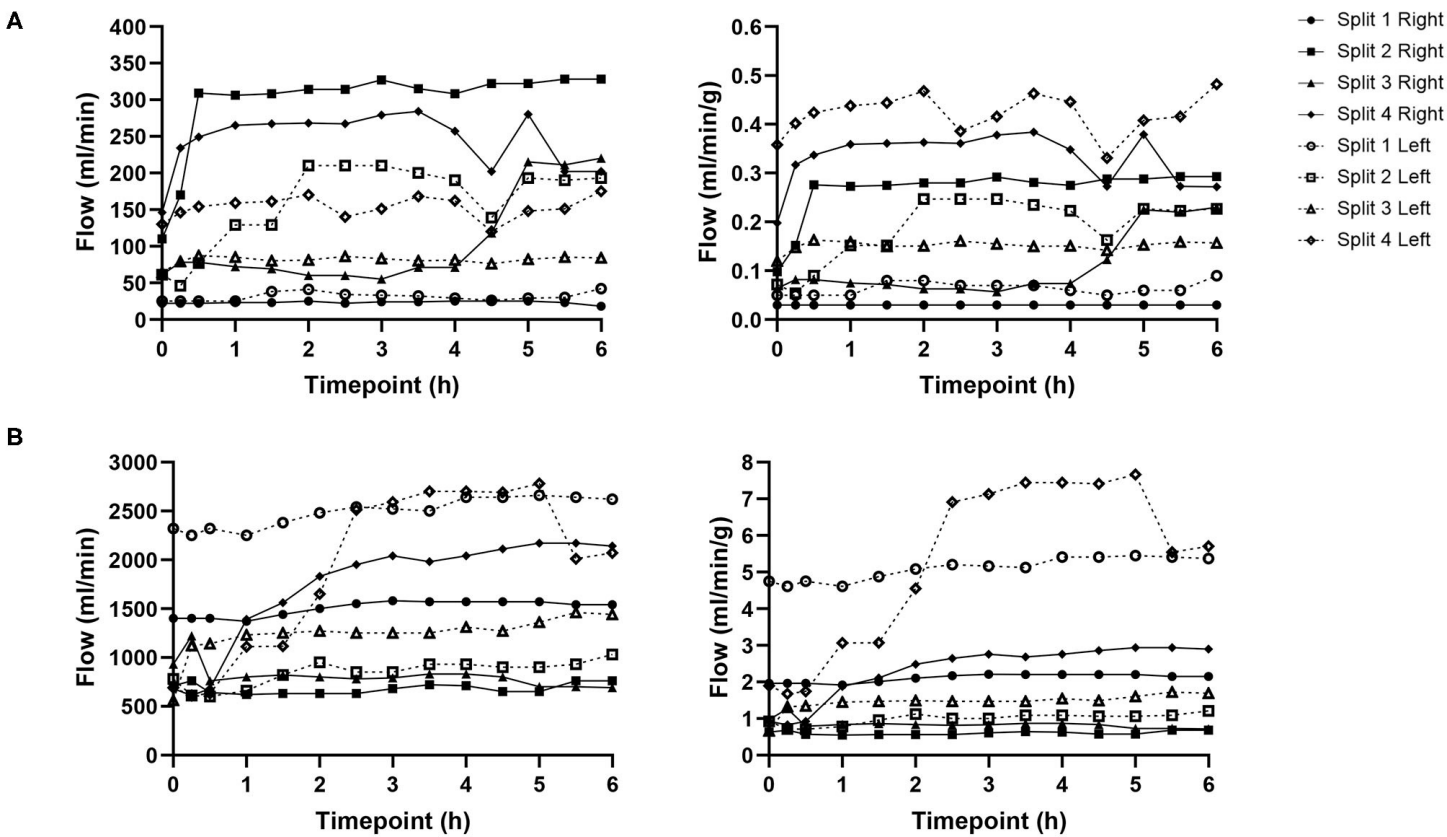
Hepatic artery **(A)** and portal vein **(B)** flows for each individual split lobe over 6 h of end-ischaemic normothermic machine perfusion.

Hepatic artery and portal venous flows were relatively consistent in both left and right lobes with a trend toward an increase in flow rate as the perfusion experiment progressed (Figure 5). Differences in flow rates for hepatic artery and portal vein were statistically insignificant across all time points.

### Liver Histology

**Figure 6:**
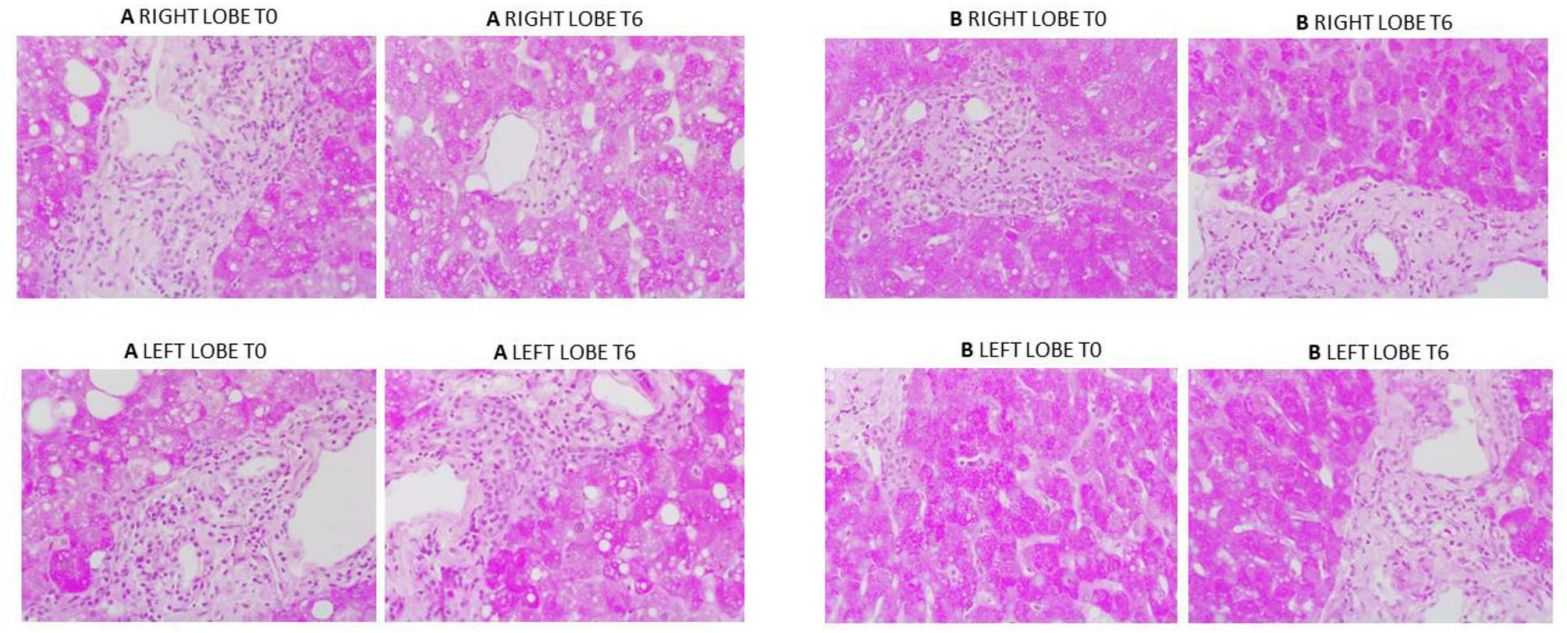
PAS staining from two representative perfusion experiments. **(A)** (from liver case number 2) shows mild to moderate macrovesicular steatosis, portal inflammatory cell infiltration and patchy PAS staining which was the same at commencement and end of perfusion. **(B)** (from liver case number 4) shows a liver with evenly distributed PAS staining throughout. Again, a degree of portal inflammation was seen. **T0**: pre-perfusion, **T6**: end of perfusion.

Overall histological analysis showed that both liver lobes behaved in a similar manner throughout the perfusions. Lobar architecture was well-preserved in both pre- and post-perfusion biopsies. Some variable centrilobular necrosis was observed at both time points. However, this was present to the same degree before and during NMLP. All biopsies were PAS positive with staining showing a range of intensity from mild to strong and ranging from even distribution throughout the parenchyma to patchy. Whatever the variability between liver, each of the paired lobes showed strong similarity, with no change throughout the perfusion. In addition, patterns of histology did not correlate with any parameters of functional assessment. Only one liver showed evidence of significant macrovesicular steatosis which did not change between lobes or throughout the perfusion (Figure 6).

## Discussion

Several studies have demonstrated that NMLP enables the functional recovery of discarded donor livers and provides a window of opportunity for viability assessment prior to transplantation. However, results are compounded by small sample size as a consequence of the limited availability of discarded donor livers for research, further compounded by the inherent differences between donor livers. This limits comparability between groups of individual livers.

Splitting of the donor liver is a well-known procedure in liver transplantation to optimize the use of grafts (9). Human split liver machine perfusion data in the normothermic setting is limited to case reports (14, 15). With this in mind, we sought to adapt the split liver technique to the normothermic machine perfusion model with the aim of providing a more robust perfusion protocol with each liver providing its own internal control. Our results show that split livers recover functionality and perform similarly to each other when subjected NMLP. The development of a split liver model to *ex situ* end-ischaemic NMLP, therefore, has the potential to provide a platform for suitable comparative controls for the investigation and assessment of therapeutic interventions prior to being subjected to the rigor of a clinical trial. Research published previously by our group allowed for an initial four-hour window of NMLP before a full viability assessment of the perfused organ is carried out (11). This period is important in the setting of split liver machine perfusion for two reasons. First and foremost, it will allow for the functional recovery of both lobes following a variable period of cold ischaemia. Secondly, it will enable the investigator to assess and monitor differences in the parameters and performance of the two lobes prior to therapeutic intervention.

The model described in the paper was developed solely for experimental purposes without considerations for clinical adoption. The inflow portal venous anatomy was without variations which allowed us to develop a standardized approached used in all livers. As the outflow from the liver was on free drainage to the reservoir, the site of caval division did not have any impact on function. In view of the open circuit perfusion system, the liver outflow veins (including segmental veins draining into the middle hepatic vein) did not require any reconstruction as the blood was freely drained and collected in the device reservoir. As such, hepatic veins anatomy and inferior vena cava accessories did not influence the surgical technique or the perfusion parameters. The situation would be different if a closed circuit was being used or if clinical use of these segmental grafts was under consideration.

The incorporation of NMLP to the split liver model has the benefit of enabling liver functional assessment under near-physiological conditions. The use of an artificial acellular hemoglobin-based perfusion fluid also has its advantages. Unlike blood products (derived from various donors), it has low immunogenicity and may therefore be of greater value in the study of liver-specific immune cell populations and mechanistic studies (12). Secondly, while the utility of blood products as perfusion fluid is limited to physiological temperatures, Hemopure can be deployed as a perfusion fluid under hypothermic conditions (12). Subsequently, our proposed split liver model can be applied to a range of perfusion temperatures.

It must be noted that there are limitations to the design of this perfusion model. First, splitting of the liver requires considerable surgical expertise and increases the cold ischaemia time beyond what is usual for the preparation of a whole liver for machine perfusion. Secondly, due to the liver's anatomy, the right lobe is invariably larger than the left lobe and therefore they cannot be split into entities of equal mass without compromising blood supply. Furthermore, the shorter and smaller caliber of hepatic artery and portal vein branches makes them much more difficult to cannulate. We attempted to circumvent this issue by retaining the main trunk of the vessel with one lobe while performing vascular reconstructions on the other, using donor iliac vessels or larger caliber sections from the proximal celiac trunk or splenic artery (if included in the specimen). In all experiments this provided the additional length for safe cannulation. However, this technique further increases the cold ischaemia time of the liver and would also require the appropriate surgical expertise for a robust reconstruction that would not compromise lobar perfusion. All these aforementioned factors may lead to differences in the perfusion characteristics of each lobe and must be considered. Finally, not all livers can be split. This is often a result of variations in arterial anatomy due to branching patterns which would compromise the inflow of a segment of the lobe if divided.

We acknowledge that the data presented in this manuscript is from a small number of human donor livers. Nevertheless, the results of this proof-of-concept study indicate that trends in the functional recovery and metabolic parameters between lobes were very similar and may therefore prove useful in the assessment of responses to therapeutic interventions in the pre-clinical normothermic setting. Additionally, this model addresses the variability that exists between individual donor livers by enabling the same donor liver to be placed in the treatment and control groups. This also maximizes use of resources as one donor liver for pre-clinical studies.

Other benefits observed during these experiments came from the use of a perfusion machine with an open circuit. These included: facilitated manipulation of the graft for direct visualization, direct visualization of and access to graft outflow and superior access to graft for direct therapeutic interventions when compared to closed circuit alternatives.

## Conclusion

Liver splitting is a feasible model for providing comparative controls for pre-clinical normothermic machine perfusion research. Further work should involve the optimisation of this protocol to minimize the need for surgical expertise when preparing the liver for machine perfusion as well as its application to other perfusion modalities, namely, hypothermic and sub-normothermic perfusion. This novel split liver model can be tested for cellular therapies to investigated cellular phenotype and lineage changes and future pharmacological interventions of donor liver before implantation.

## Data Availability Statement

The raw data supporting the conclusions of this article will be made available by the authors, without undue reservation.

## Ethics Statement

The studies involving human participants were reviewed and approved by the London-Surrey Borders National Research Ethics Service (Reference Number 13/LO/1926) and the NHSBT Ethics Committee (Reference Number 06/Q702/61). Informed consent for research use of donor organs was obtained by specialist nurses in organ donation from the donor’s next of kin during informed consent for organ donation.

## Author Contributions

JA, YB, LW, and SA designed the study and established the methodology. JA, LW, DO-B, and VR conducted the experiments. JA and LW collected the data. DM and HM supervised the liver splitting procedure. JA performed the data analysis and drafted the manuscript. SA and GR reviewed the histological specimens. YO, MP, HM, and DM contributed to critical revision and editing of the final manuscript. All authors approved the final version of this manuscript.

## Conflict of Interest

JA is a clinical research fellow at the Queen Elizabeth Hospital in Birmingham and employed by University Hospitals Birmingham. The remaining authors declare that the research was conducted in the absence of any commercial or financial relationships that could be construed as a potential conflict of interest.

## Abbreviations

BD: Bile duct
DBD: Donor after Brainstem Death
DCD: Donor after Cardiac Death
CHA: Common Hepatic Artery
LHV: Left Hepatic Vein
LPV: Left Branch of Portal Vein
LL: Left Lobe
MHV: Middle Hepatic Vein
NHSBT: National Health Service Blood and Transplant
NMLP: Normothermic Machine Liver Perfusion
PAS: Periodic Acid Schiff
PV: Portal Vein
RHA: Right Hepatic Artery
RHV: Right Hepatic Vein
RL: Right Lobe
RPV: Right Branch of Portal Vein
T0: Start of Perfusion
T6: End of Perfusion after 6 h
VC: Vena Cava.
