# Supplementary material for: Ex situ Normothermic Split Liver Machine Perfusion: Protocol for Robust Comparative Controls in Liver Function Assessment Suitable for Evaluation of Novel Therapeutic Interventions in the Pre-clinical Setting
Source: Front Surg. 2021 Feb 17;8:627332. doi: 10.3389/fsurg.2021.627332 (PMC7925992; doi:10.3389/fsurg.2021.627332)
Supplement: Supplementary file 1 [file Table_1.DOCX]

**SUPPLEMENTARY MATERIAL**

Ex Situ Normothermic Split Liver Machine Perfusion: Protocol for Robust Comparative Controls in Liver Function Assessment suitable for the evaluation of novel therapeutic interventions in the preclinical setting

**Authors:** J A Attard^1,2,3^, D Osei-Bordom^1,2^,Y Boteon^1,2,3^, L Wallace^1,2^, , V Ronca^1,2^, G Reynolds^1,2^, T Perera^3^, Y Htun Oo^1,2,3,4^, H Mergental^1,2,3^, D F Mirza^3^, S C Afford^1,2^.

^1^ NIHR Birmingham Biomedical Research Centre, University Hospitals Birmingham NHS Foundation Trust and University of Birmingham, Edgbaston, Birmingham, UK
^2^ Centre for Liver and Gastrointestinal Research, Institute of Immunology and Immunotherapy, University of Birmingham, Edgbaston, Birmingham UK.

^3^ Liver Unit, Queen Elizabeth Hospital, University Hospitals Birmingham NHS Foundation Trust, Edgbaston, Birmingham, UK

^4^Centre for Rare Disease, European Reference Network Centre, RARE LIVER

**Matherials and Method - Perfusate fluid components**

- 3 units (250mL/unit) HBOC-201
- 1000 mL 5% w/v human albumin solution (Alburex 5, CSL Behring GmbH, Germany)
- 10,000 IU heparin (Wockhardt, UK)
- 30mL 8.4 % sodium bicarbonate 8.4% (B. Braun Medical Limited, UK)
- 10 mL 10% calcium gluconate
- 500 mg vancomycin (Wockhardt, UK)
- 60 mg gentamicin (Cidomycin, Sanofi, UK)
- 50 mL 10% v/v Aminoplasmal (B.Braun Medical Limited, UK)
- 0.2 mL Cernevit (Baxter Healthcare Ltd., UK)
- 0.1 mg phytomenadione (Konakion, Roche Products Ltd, UK)
- Epoprostenol (Flolan, GlaxoSmithKline, UK, 2 µg/ml) continuous infusion at 4 - 8 mL/hour
